# An Augmented SMS Intervention to Improve Access to Antenatal CD4 Testing and ART Initiation in HIV-Infected Pregnant Women: A Cluster Randomized Trial

**DOI:** 10.1371/journal.pone.0117181

**Published:** 2015-02-18

**Authors:** Scott Dryden-Peterson, Kara Bennett, Michael D. Hughes, Adrian Veres, Oaitse John, Rosina Pradhananga, Matthew Boyer, Carolyn Brown, Bright Sakyi, Erik van Widenfelt, Koona Keapoletswe, Madisa Mine, Sikhulile Moyo, Aida Asmelash, Mark Siedner, Mompati Mmalane, Roger L. Shapiro, Shahin Lockman

**Affiliations:** 1 Division of Infectious Diseases, Brigham and Women’s Hospital, Boston, Massachusetts, United States of America; 2 Botswana Harvard AIDS Institute Partnership, Gaborone, Botswana; 3 Department of Immunology and Infectious Diseases, Harvard School of Public Health, Boston, Massachusetts, United States of America; 4 Harvard Medical School, Boston, Massachusetts, United States of America; 5 Bennett Statistical Consulting, Inc., Ballston Lake, New York, United States of America; 6 Department of Biostatistics, Harvard School of Public Health, Boston, Massachusetts, United States of America; 7 Yale School of Public Health, New Haven, Connecticut, United States of America; 8 Joan C. Edwards School of Medicine, Marshall University, Huntington, West Virginia, United States of America; 9 John’s Hopkins Bloomberg School of Public Health, Baltimore, Maryland, United States of America; 10 Ministry of Health, Gaborone, Botswana; 11 Division of Infectious Diseases, Massachusetts General Hospital, Boston, Massachusetts, United States of America; 12 Division of Infectious Diseases, Beth Israel Deaconess Medical Center, Boston, Massachusetts, United States of America; Centers for Disease Control and Prevention, UNITED STATES

## Abstract

**Background:**

Less than one-third of HIV-infected pregnant women eligible for combination antiretroviral therapy (ART) globally initiate treatment prior to delivery, with lack of access to timely CD_4_ results being a principal barrier. We evaluated the effectiveness of an SMS-based intervention to improve access to timely antenatal ART.

**Methods:**

We conducted a stepped-wedge cluster randomized trial of a low-cost programmatic intervention in 20 antenatal clinics in Gaborone, Botswana. From July 2011-April 2012, 2 clinics were randomly selected every 4 weeks to receive an ongoing clinic-based educational intervention to improve CD_4_ collection and to receive CD_4_ results via an automated SMS platform with active patient tracing. CD_4_ testing before 26 weeks gestation and ART initiation before 30 weeks gestation were assessed.

**Results:**

Three-hundred-sixty-six ART-naïve women were included, 189 registering for antenatal care under Intervention and 177 under Usual Care periods. Of CD_4_-eligible women, 100 (59.2%) women under Intervention and 79 (50.6%) women under Usual Care completed CD_4_ phlebotomy before 26 weeks gestation, adjusted odds ratio (aOR, adjusted for time that a clinic initiated Intervention) 0.87 (95% confidence interval [CI]0.47–1.63, P = 0.67). The SMS-based platform reduced time to clinic receipt of CD_4_ test result from median of 16 to 6 days (P<0.001), was appreciated by clinic staff, and was associated with reduced operational cost. However, rates of ART initiation remained low, with 56 (36.4%) women registering under Intervention versus 37 (24.2%) women under Usual Care initiating ART prior to 30 weeks gestation, aOR 1.06 (95%CI 0.53–2.13, P = 0.87).

**Conclusions:**

The augmented SMS-based intervention delivered CD_4_ results more rapidly and efficiently, and this type of SMS-based results delivery platform may be useful for a variety of tests and settings. However, the intervention did not appear to improve access to timely antenatal CD_4_ testing or ART initiation, as obstacles other than CD_4_ impeded ART initiation during pregnancy.

## Introduction

To achieve the goal of elimination of mother-to-child transmission of HIV (MTCT), timely access to combination antiretroviral therapy (ART) is vital for the 1.4 million HIV-infected women who become pregnant annually [1]. When started before the third trimester of pregnancy ART reduces the risk of MTCT to 1–2% [2–10], even in breastfeeding populations [2]. ART is now recommended by the World Health Organization (WHO) for both maternal health and for MTCT prevention for all pregnant and breastfeeding women [11].

Botswana and other countries in the region have made remarkable progress at improving access to HIV testing and single-drug prevention of MTCT strategies [1,12]; however, access to timely antenatal ART has lagged. Less than one-third of treatment-eligible women initiate ART prior to delivery, with early antenatal CD4 testing being identified as a substantial barrier [13–16]. Although no longer a requirement for starting ART in programs offering a life-long efavirenz-based ART regimens to pregnant women independent of disease stage (WHO Option B+) [17], drawing a CD4 prior to ART initiation remains vital in countries such as Botswana where CD4 testing is needed to guide whether ART should stop after MTCT prevention or continue for maternal health (WHO Option B). In these settings delayed receipt of a CD4 test result may be a bottleneck to the timely start of ART in pregnancy. In addition, timely CD4 enumeration is important to inform decisions for prophylaxis and evaluation for opportunistic conditions.

Through key informant interviews and analysis of the prevention of MTCT cascade in a large birth registry[18], we identified three principal obstacles for antenatal ART initiation in Botswana: 1) difficulty completing CD4 phlebotomy during the first antenatal visit due to limitations in supplies, staffing, and specimen transportation, 2) delay and unpredictability in the return for CD4 test results from the central laboratory, and 3) challenges locating ART-eligible women after receipt of CD4 test results. These treatment-eligible women, with CD4+ cell counts less than 350 cells/μL, account for greater than 80% of infant infections and an equal proportion of maternal deaths [19]. Timely CD4 testing and initiation of ART can ameliorate both risks[5,6,20,21].

Leveraging the potential of mobile health technology (mHealth) [22–25], we developed a low-cost clinic-based intervention to address each of these three obstacles, including 1) participatory provider education reinforcing protocols for CD4 testing, 2) an open source, automated platform permitting monitoring and delivery of CD4 results via short message service (SMS) between central laboratory and peripheral clinics, and 3) support for tracing women eligible for ART initiation. We sought to evaluate whether this intervention improved timely CD4 testing and ART initiation among HIV-infected, ART-naïve women in Botswana.

## Methods

### Ethics Statement

Officials and clinicians in charge of participating clinics provided written documentation of permission. A waiver of informed consent was obtained to abstract routine clinical information from patient obstetric and maternity records. All women participating in validation interviews by study staff provided written informed consent. The study was reviewed and approved by the Botswana Health Research Development Committee and the Institutional Review Board of the Harvard School of Public Health. The trial clinicaltrials.gov registration number is NCT01836003. Registration occurred after trial completion due to misunderstanding as to whether a programmatic intervention should be registered and future trials would be registered. The protocol for this trial and supporting CONSORT checklist are available as supporting information; see S1 CONSORT Checklist and S1 Protocol.

### Study Setting

The 20 highest volume antenatal clinics in greater Gaborone were included in this project, which was named Tokafatso (‘improvement’ in Setswana, the local language). At the start of the project in July 2011, each of these clinics provided antenatal care, onsite HIV testing and counseling, and phlebotomy (or referral for phlebotomy) for CD4 enumeration. CD4 testing and most deliveries occurred at the national referral hospital in Gaborone. At study start, clinics received CD4 results via hand-delivered paper reports (9 clinics), electronic medical record (EMR, 3 clinics), or travel to and transcription from an EMR-enabled clinic (8 clinics). Six clinics had onsite ART services (not integrated with antenatal care activities), and 14 clinics referred offsite for ART initiation.

At the start of the project, women with a CD4 count of 250 cells/μL or less (or WHO stage III/IV) were eligible for ART. Women with a CD4 count greater than 250 cells/μL received zidovudine from 28 weeks gestation and single-dose nevirapine in labor [26]. A pilot program was in place in 5 study clinics, which expanded ART eligibility to all pregnant women regardless of CD4 count. In this program, CD4 testing was required to direct ART regimen choice and determine whether treatment would be continued after delivery. During the course of the project, new national guidelines were released and gradually adopted by clinic staff beginning in late 2012. These revised guidelines recommended ART for all HIV-infected women during pregnancy and indefinite ART for women with a CD4 count of 350 cells/μL or less (or WHO stage III/IV) [27,28]. While a CD4 result was not required for ART initiation following this revision, clinicians generally still performed and waited for CD4 count results before starting ART.

### Study Design

We conducted a cluster randomized study utilizing a ‘stepped wedge’ design [29,30]. This design was selected to ensure that all clinics could benefit from the Tokafatso intervention, permit adjustment for time effects given evolving prevention of MTCT programs, and enable effective implementation by a single study team. During the course of ten 4-week blocks between July 2011 and April 2012, all antenatal care clinics (ANCs) received the SMS-based intervention, but the order of implementation was randomized. Clinics and the study team were blinded to randomized order. One week prior to the start of a block, a randomization envelope was opened revealing the names of the 2 clinics to receive the intervention during that block. Implementation was scheduled as close to the first day of the block as practicable. Once implementation occurred, the intervention remained in effect.

**Fig 1:**
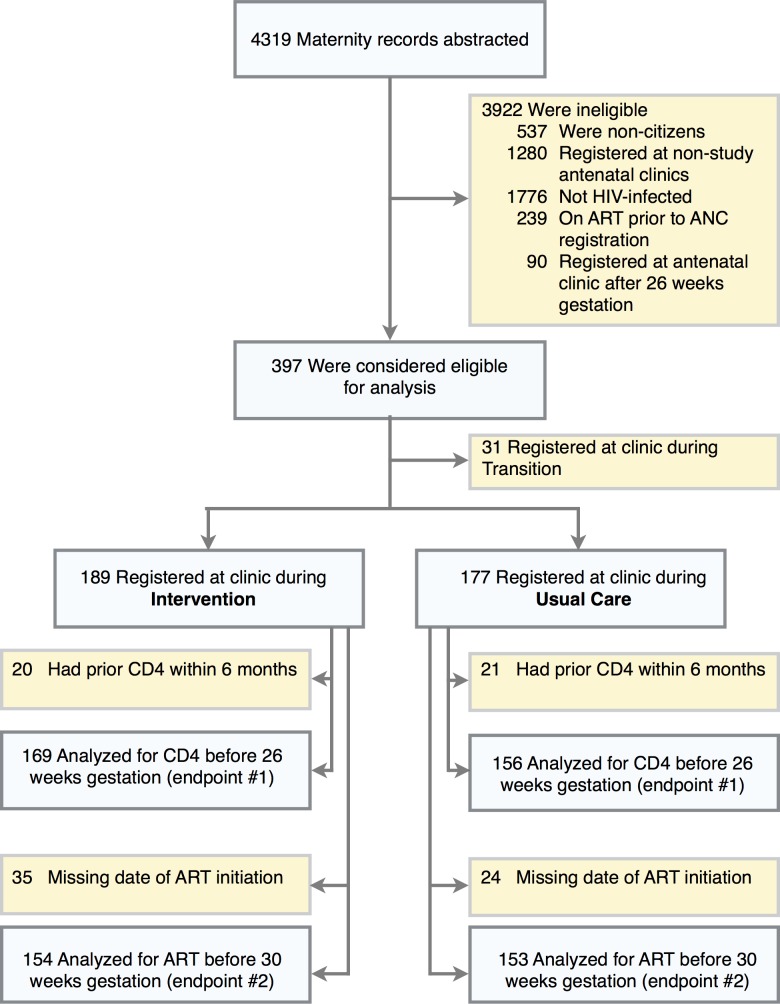
Abstraction, eligibility, and analysis. Women could have more than one reason for ineligibility for the analysis.

**Fig 2:**
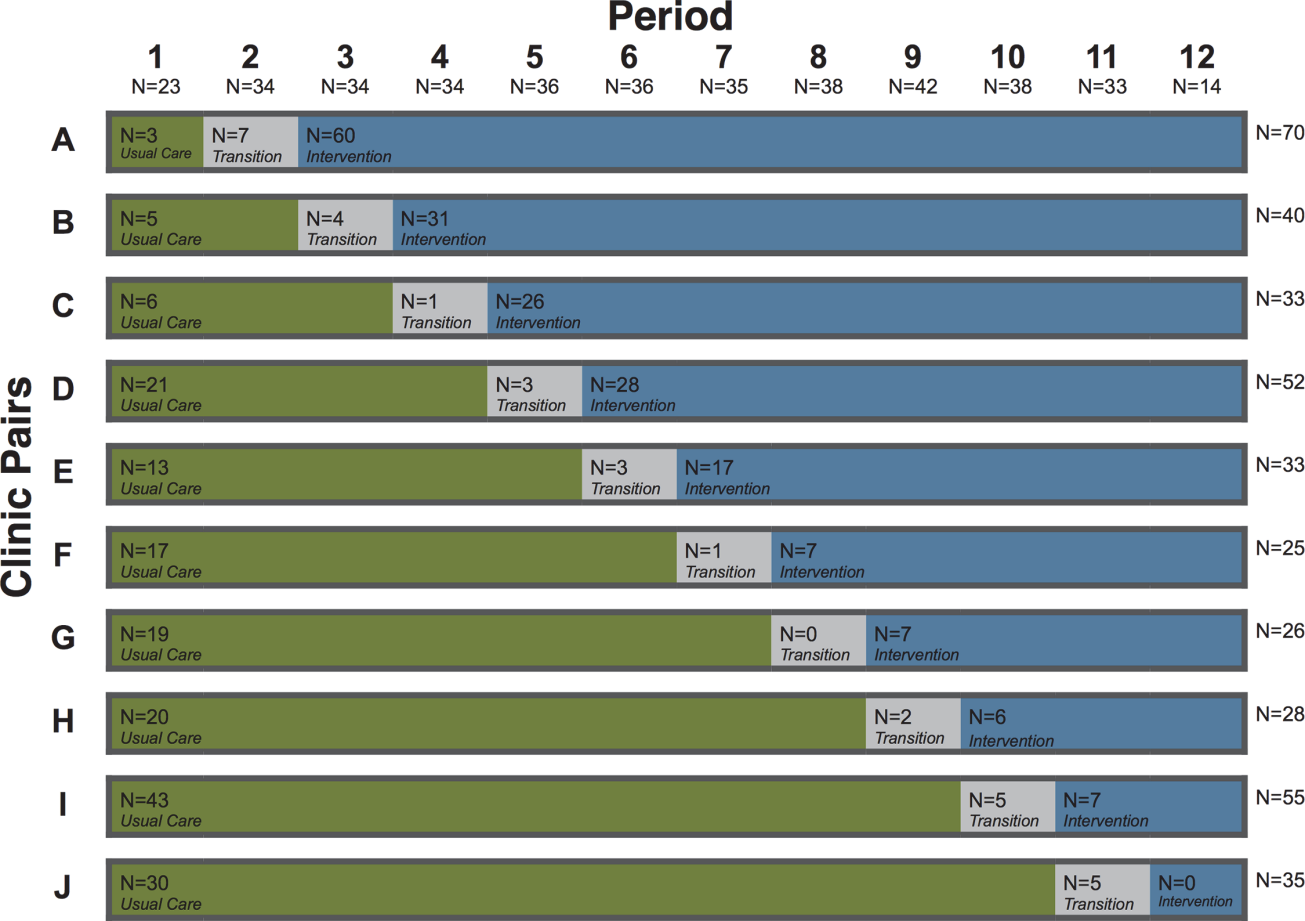
Stepped-wedge implementation of the intervention by study period.

A 12-week lead-in period prior to implementation at the first clinics was planned. However, a generalized public service strike leading to restricted health services limited this baseline period to 3 weeks. A follow-up period of 4-weeks was included after implementation in all clinics for capture of additional pregnancies (Figs. 1 and 2).

Two randomly selected clinics (one pair) received the SMS-based intervention every 4 weeks from Period 2 to Period 11. Note: The sample sizes for each period and each clinic pair are provided along the horizontal and vertical axes, respectively. The sample sizes for each clinic pair are recorded for Usual Care, Transition, and Intervention in the colored bars.

### Endpoints and Ascertainment

ART-naïve, HIV-infected Botswana citizens registering at a participating antenatal clinic prior to 26 weeks gestation were included in the primary analysis. The primary study endpoints were the proportion of HIV-infected, ART-naïve pregnant women a) completing phlebotomy for CD4 enumeration by 26 weeks gestation and b) initiating ART by 30 weeks gestation. For analyses related to CD4 testing, women with a CD4 result within 6 months of antenatal clinic registration were excluded as these women may not have been eligible for retesting.

Study endpoints were assessed through the abstraction of data from routine obstetric, maternity, and laboratory records at Princess Marina Hospital, the principal delivery ward for the study antenatal clinics. Records of deliveries from June 2011 through October 2012 were reviewed. Gestational age was estimated from the date of the last normal menstrual period as recorded in the obstetric record. Gestational age at delivery was imputed from the population median of eligible women for records with missing or unlikely (gestational age at delivery less than 22 or more than 45 weeks gestation) dates.

For purposes of the analysis, a women was defined as being in the Intervention or Usual Care period on the basis of whether the clinic was under usual care the intervention or usual care at the time of her registration for antenatal care. Women first presenting for antenatal care during the initial 4 weeks of the clinic receiving the intervention were assigned to Transition period and not included in the primary analysis.

To assess accuracy and completeness of the abstracted information and to better understand barriers to timely initiation of ART, a convenience sample on consenting HIV-infected women delivering without a recorded CD4 result, or with a CD4 count of 250 cells/μL or less but not on ART, were interviewed.

As designed, the study had estimated 90% power to detect an absolute 15% improvement in CD4 testing by 26 weeks gestation (estimated baseline 36%) and a 35% improvement in rate of ART initiation before 30 weeks gestation (estimated baseline 33%).

### Intervention

The Tokafatso intervention included an improvement package of augmented services around a novel SMS-based platform for CD4 result distribution to antenatal clinics (Table 1). The package included the following:

**Table 1:** Required steps to receive antenatal ART and description of Tokafatso intervention activities.

Cascade Step	Tokafatso Intervention Activities
Antenatal clinic registration	None.
HIV testing	Provision of HIV test kits through loans during periods of supply outage.
CD4 phlebotomy	Educational session (1–2 hours) and twice-monthly contact with clinic to support early CD4 phlebotomy.
	Provision of CD4 phlebotomy supplies through loans during periods of supply outage.
	Facilitated solutions to transportation challenges to increase number of clinic days when CD4 phlebotomy could be performed.
	Central monitoring of CD4 testing frequency and outreach to clinics with lower than expected test frequency.
Laboratory processing of CD4 specimen	Longitudinal monitoring of time to CD4 result permitting real-time inquiries regarding delayed results.
Clinic receipt of CD4 result	Novel SMS-based platform that delivered results directly to a printer in the antenatal clinic (typically at desk of midwife) and provided chain-of-custody.
Patient receipt of CD4 results	For each CD4 ≤ 250 cells/μL the antenatal clinic was contacted and provided with airtime to call patient and request that she come to the clinic to receive results and counseling.
ART clinic registration and baseline laboratory testing	None.
ART adherence counseling, typically with a friend or family member serving as an adherence partner	None.
ART clinician evaluation and pick-up of ART prescription	None.

Note: During the course of the project, receipt of a CD4 result became no longer formally required prior to ART initiation. However, all clinic staff reported that they would not refer a patient to the ART clinic without a CD4 count result. ART, combination antiretroviral therapy.

**Fig 3:**
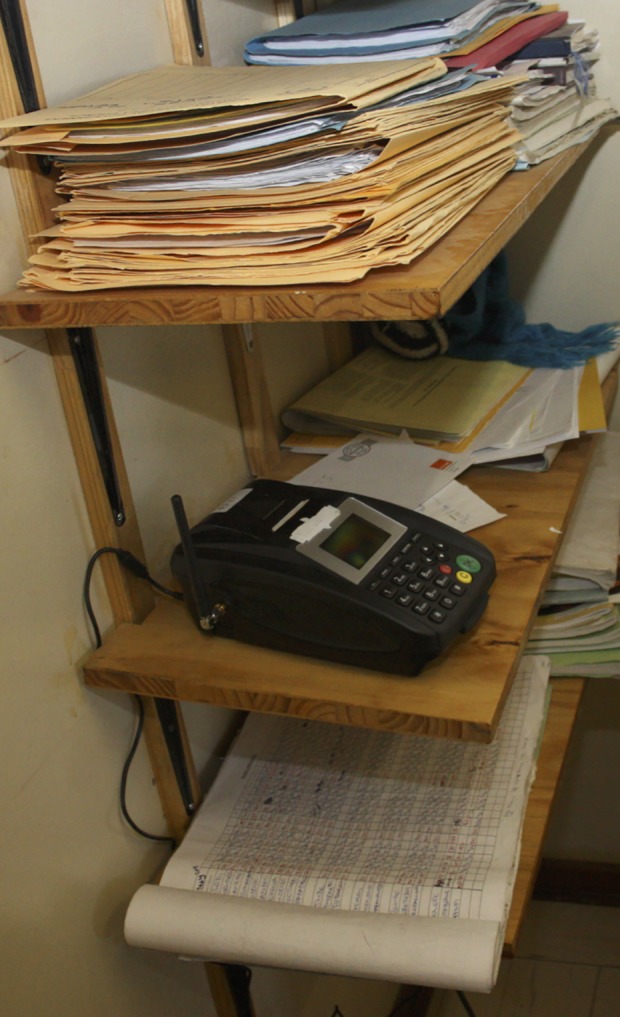
Mobile SMS printer in use. Printer is installed in an exam room of a study antenatal clinic.

- A 1–2 hour participatory session for clinic staff emphasizing the importance of timely CD4 testing and ART initiation, and developing solutions to identified challenges to clinic performance (e.g. result delivery, transportation, supply management, patient tracing, staff shortages).
- An automated SMS-based CD4 result platform that wirelessly distributed CD4 results to portable SMS-enabled thermal printers (iBacsTel Electronics, Cardiff, United Kingdom) located in each of the study antenatal clinics. The software was authored in Python (Python Software Foundation, Wilmington, Delaware) over a MySQL database (Oracle, Redwood City, California) using Django (Django Software Foundation, Lawrence, Kansas). The system directly collected results from flow cytometer (FACSCalibur, BD, Franklin Lakes, New Jersey) output files and integrated demographics and validation status from electronic records. The system then sent valid results via an SMS gateway (Kannel, The Kannel Group, Helsinki, Finland) to printers in referring antenatal clinics. Results were printed as soon as results were available. Clinic receipt of results was confirmed centrally via SMS, and modules in system permitted central monitoring of parameters of clinic and laboratory performance (e.g. alerts with testing frequency fell below expected ranges or when laboratory validation results delayed). Clinic staff was required to ensure printers were adequately charged and confirm that the printer had adequate paper. The Tokafatso system software is available of free of charge (https://github.com/adrianveres/tokafatso).
- Longitudinal support by a study team member to antenatal clinics, including educating new clinic staff, supporting SMS printers, assisting with supply outages (e.g. of HIV or CD4 testing supplies), and proactively contacting clinics and the laboratory during periods of suspected underperformance (e.g. responding to automated alerts). In addition, the study personnel also reviewed ART-eligible cases with antenatal clinic staff, and provided airtime to staff at antenatal clinics to contact all of these patients via mobile phone. The study team obtained confirmation of successful patient contact by the antenatal clinic (see Fig. 3).

### Operational Cost Estimation

The operational cost of delivering a CD4 result was estimated for the Tokafatso SMS-based platform and for the three other modalities utilized for result distribution to the greater Gaborone antenatal clinics. Operational costs of the Tokafatso platform were estimated through review of time logs, transportation costs, and expenditures for SMS messages. Through interviews with clinic staff we determined, for each cadre of the clinic staff, the monthly time spent in CD4 result retrieval (reviewing paper or electronic records to find results), transport to locations to retrieve CD4 results, and support of computerized systems. Salaries for each cadre were estimated using representative salaries from the Botswana Harvard AIDS Institute in Botswana. Fuel and vehicle costs were estimated at 1.75 pula per kilometer. Fixed infrastructure costs, including licensing and support fees to the provider of the government electronic medical record system (MEDITECH, Westwood, Massachusetts), were not included. Cost per CD4 result delivered was estimated from the total monthly cost related to CD4 delivery, total number of CD4 results delivered, and the proportion of these results that were antenatal CD4 results.

### Statistical Analysis

Comparisons of the study endpoints were performed utilizing general estimating equations with adjustment for temporal effects and clustering at the clinic level following standard methodology for stepped-wedge trials [30]. Small sample size precluded inclusion of individual 4-week blocks, consequently temporal efforts were modeled using 12-week periods. The primary models (Model A) accounted for clustering of participants within clinics and were adjusted for the time that a clinic started the intervention. Findings in the primary models were assessed for sensitivity to imputation strategy used for missing gestational ages, alternate group assignment definitions, significant univariate predictors of endpoints, exclusion of individual clinics, and different methods of modeling temporal effects. In addition, a secondary model (Model B) including all factors identified as significantly associated with the endpoint was also fit to permit adjustment for possible confounding. Analyses of time to testing or ART initiation were conducted using marginal Cox models that incorporated the clinic-level clustering through the use of robust sandwich covariance estimates [31].

Statistical analyses were performed with the use of the SAS statistical package, version 9.2 (SAS Institute, Cary, North Carolina). All tests were two-tailed and P-values of less than 0.05 were considered statistically significant.

## Results

### Study Clinics and Deliveries

A total of 19 of the 20 randomized antenatal clinics received the intervention. One antenatal clinic had begun sending CD4 specimens to a different laboratory for analysis just prior to receipt of the intervention. As the clinic could not receive the SMS and patient tracing components of the intervention, data from patients registering at this clinic after the change in CD4 testing were excluded.

During the study period, 2502 Botswana citizens registering at one of the study antenatal clinics delivered at the principal maternity ward for these clinics. Of these, 726 were HIV-infected (29.0%), 1769 (70.7%) were HIV-negative, and 7 (0.3%) had unknown HIV status. A total of 239 (32.9%) HIV-infected women were already on ART prior to receiving antenatal care. Of the remaining 487 ART-naïve women, 397 presenting for antenatal care prior to 26 weeks gestation without a CD4 count in the prior 6 months were included in the analyses. Of these women 177 (44.6%) registered under Usual Care, 189 (47.6%) under Intervention, and 31 (7.8%) during Transition. The median gestational age at antenatal clinic registration was 18 weeks (Intervention 18 weeks, Usual Care 17 weeks, P = 0.30). The comparison groups were well balanced in baseline characteristics (Table 2), except that women registering under Intervention had less education and were more likely to receive care at a clinic with an on-site ART clinic and early adoption of universal ART (WHO option B).

**Table 2:** Characteristics of ART-naïve, HIV-infected pregnant women and their antenatal clinics by study group.

Characteristic		Intervention N = 189	Usual Care N = 177
Maternal age (years), median (quartiles)		28 (25, 33)	29 (26, 33)
Marital status, no. (%)			
	Married	17 (9%)	17 (10%)
	Single/Divorced/Widowed	168 (89%)	157 (89%)
	Unknown	4 (2%)	3 (2%)
Education, no. (%)			
	Primary or none	12 (6%)	16 (9%)
	Secondary	157 (83%)	130 (73%)
	University	15 (8%)	29 (16%)
	Unknown	5 (3%)	2 (1%)
Imputed date of LNMP (missing or out-of-range),^a^ no. (%)		16 (8%)	10 (6%)
Gestational age (weeks) at ANC registration, median (quartiles)		18 (14, 21)	17 (14, 21)
Gestational age (weeks) at delivery, median (quartiles)		38 (36, 40)	38 (35, 40)
Premature delivery (< 37 weeks), no. (%)		59 (31%)	61 (34%)
Timing of positive HIV test, no. (%)			
	Prior to pregnancy	53 (28%)	58(33%)
	During pregnancy	131 (69%)	115 (65%)
	After pregnancy/Unknown	5 (3%)	4 (2%)
CD4 count, no. (%)			
	≤ 250 cells/μL	34 (18%)	32 (18%)
	> 250 cells/μL	113 (60%)	99 (56%)
	Missing	42 (22%)	46 (26%)
ANC with on-site ART clinic, no. (%)		89 (47%)	48 (27%)
ANC with on-site access to EMR, no. (%)		25 (13%)	30 (17%)
ANC received early implementation of universal ART, no. (%) ^b^		78 (41%)	19 (11%)

Note: IQR, interquartile range; LNMP, last normal menstrual period; ANC, antenatal clinic; ART, combination antiretroviral therapy; EMR, electronic medical record.

^a^ Women with calculated gestational age less than 22 weeks or greater than 45 weeks were considered likely out-of-range.

^b^ Implementation of universal ART (WHO option B) prior to November 2011.

### Primary Analyses

**CD4 draw**. Of ART-naïve, CD4-eligible women, 100 (59.2%) women registering at an ANC during Intervention and 79 (50.6%) women registering during Usual Care completed CD4 phlebotomy prior to 26 weeks gestation. There was no significant difference between groups in the primary analysis model (Model A) adjusting for clustering and temporal effects (adjusted odds ratio (aOR) 0.87, 95% confidence interval [CI] 0.47–1.63, P = 0.67). Findings were similar after additional adjustment (Model B) for possible confounders (Table 3). There were significant temporal effects in both models (P<0.001) with overall improvement in the proportion of women undergoing timely CD4 testing during the study period. During the first 3 months of the study period—immediately following a protracted public employee strike—performance under Usual Care clinics was poorer with 26.5% of women having CD4 by 26 weeks compared with 69.3% during later periods. Registration to antenatal care later in pregnancy, unmarried status, new HIV diagnosis in pregnancy, and presentation to a clinic with early implementation of universal ART were each significantly associated with decreased odds of CD4 by 26 weeks gestation.

**Table 3:** Primary endpoints, timely CD4 phlebotomy and HAART initiation in eligible women.

		CD4 phlebotomy before 26 weeks gestation		ART initiation before 30 weeks gestation	
		success/eligible (percent)		success/eligible (percent)	
		Intervention	Usual Care	Intervention	Usual Care
**Success by period**					
	Periods 1–3	0/4 (0%)	18/68 (26%)	0/5 (0%)	10/65 (15%)
	Periods 4–6	17/36 (47%)	38 /51 (75%)	9/30 (30%)	18/52 (35%)
	Periods 7–9	37/61 (61%)	22/36 (61%)	20/60 (33%)	8/34 (24%)
	Periods 10–12	46 /68 (68%)	1/1 (100%)	27/59 (46%)	1/2 (50%)
	Total	100/169 (59%)	79/156 (51%)	56/154 (36%)	37/153 (24%)
		**Odds Ratio** (95% CI)	**P-value** ^a^	**Odds Ratio** (95% CI)	**P-value** ^a^
**Overall Effect Estimates**					
Women registering during Intervention versus Usual Care periods					
	Model A: adjusted for study period only	0.87 (0.47, 1.63)	0.67	1.06 (0.53, 2.13)	0.87
	Model B: adjusted for study period and potential confounders	1.20 ^b^ (0.52, 2.78)	0.66	1.01 ^c^ (0.46, 2.22)	0.98

Note: ART, combination antiretroviral therapy; 95% CI, 95% confidence interval.

^a^ Wald statistic.

^b^ In addition to study period, adjusted for factors significant in univariate analysis: gestational age at antenatal registration, maternal marital status, maternal level of education, timing of maternal HIV diagnosis (prior or during current pregnancy), whether antenatal clinic implemented universal ART (WHO option B) prior to November 2011.

^c^ In addition to study period, adjusted for factors significant in univariate analysis: gestational age at antenatal registration, maternal marital status, and maternal age.

**ART Initiation**. Among ART-naïve women, 56 (36.4%) women registering under Intervention and 37 (24.2%) women registering under Usual Care initiated ART prior to 30 weeks gestation. There were no significant differences in ART initiation prior to 30 weeks between Intervention and Usual Care in the primary analysis model (Model A)—aOR 1.06 (95% CI 0.53–2.13, P = 0.87). Additional adjustment for possible confounders (Model B) produced similar results. There was also no significant difference in ART initiation prior to delivery between the study groups, aOR 1.43 (95% CI 0.85–2.41, P = 0.18).

**Impact on Specific Elements of the Prevention of MTCT Cascade**. Rates of HIV testing were high under both Intervention (99.7%) and Usual Care (99.6%), however rates of CD4 testing and ART initiation remained low.

**Timing of CD4 Phlebotomy**. HIV-infected women eligible for CD4 enumeration were no more likely to have a CD4 drawn within 1 day of registration if their clinic was under Intervention or operating under Usual Care, 19.6% and 16.4%, respectively (P = 0.33). Time from antenatal clinic registration to CD4 testing was also not improved under Intervention versus Usual Care (adjusted Hazard Ratio [aHR] 0.69, 95%CI 0.48–1.00, P = 0.05). Women registering under the Intervention actually had significantly decreased odds of having a CD4 draw prior to delivery compared with those registering during Usual Care periods (aOR 0.45, 95% CI 0.21–0.94, P = 0.035).

**Receipt of CD4 Results**. Results from CD4 testing were obtained faster under the intervention. Prior to implementation of the SMS-based platform for CD4 delivery, clinics obtaining paper CD4 results reported a typical turn-around time of 17 days from sample draw to results receipt at the clinic (range 7–35 days). Clinics with EMR access reported turn-around of 7 days (range 3–14 days), and clinics needing to travel to an EMR-enabled clinic reported 14 days (range 7–28 days). Weighting by proportion of results sent by each method, at baseline results took a median of 16 days to reach clinics after CD4 phlebotomy. Under the SMS-based platform, results were received by the antenatal clinic in a median of 6 days (interquartile range [IQR] 3–7 days), significantly faster that under Usual Care (P<0.001, single sample Wilcoxon). The SMS-based platform enabled more than 90% of ART-eligible women to be contacted by the antenatal clinic and counseled to present for ART within 10 days of CD4 phlebotomy.

**CD4 Phlebotomy to ART Initiation**. CD4 testing prior to 26 weeks gestation was strongly associated with ART initiation prior to 30 weeks gestation—aOR 7.48 (95% CI 3.69–15.1, P<0.001). Registration under Intervention, however, was not significantly associated with decreased time from CD4 draw to ART initiation compared with Usual Care—aHR 0.89 (95%CI 0.60–1.31, p = 0.55). Time from clinic registration to ART initiation was also not significantly different between groups in adjusted analyses (aHR 0.88 95% CI 0.56–1.37, p = 0.56).

### Operational Cost

Labor expenses represented the majority of the operational costs for the Tokafatso SMS-based platform and the existing CD4 result delivery methods employed in the study clinics. The number of staff hours (time spent by nurses, data clerks, drivers, computer technicians) required was lower utilizing the Tokafatso system compared with existing methods, 0.26 versus 0.39 staff hours per CD4 result, respectively. The Tokafatso system delivered a CD4 result for an estimated $1.98 (lower $1.82, upper $2.14) per result delivered compared with an overall cost of $2.73 (lower $2.56, upper $2.89) with standard delivery methods.

### Interviews

Twenty postpartum, HIV-infected women who had received care at one of the study antenatal clinics were approached and consented to be interviewed. Fifteen of these women were selected for interview because they had no recorded antenatal CD4 measurement. The interviews confirmed that none of these women had received CD4 results, however 10 women (67%) reported that blood had been drawn for CD4 but that they did not receive the results (clinic couldn’t locate results- 7, patient delivered prior to resulting- 2, patient couldn’t travel to clinic to collect- 1). Five (33%) women reported never having blood drawn for CD4 (verified no electronic record of CD4 draw).

Five women were selected for interview because they were eligible to start ART, but did not initiate ART during pregnancy. Of these, 3 (60%) reported that could not start ART as the clinic required them to have an adherence partner participate in counseling prior to initiation. One woman reported that the ART clinic delayed starting for reasons that were not clear to her and one women reported that she was not ready to accept treatment so soon after learning that she was HIV-infected.

Midwives and HIV lay counselors from all 19 antenatal clinics that received the intervention, and the regional prevention of MTCT coordinator, expressed that the intervention assisted them with caring for HIV-infected pregnant women. There was strongest support for SMS CD4 result platform and for the airtime provided to contact ART-eligible women. All clinics expressed that they would like the project continued.

## Discussion

In this prospective randomized trial, an augmented SMS-based programmatic intervention in antenatal clinics did not improve timely CD4 testing or ART initiation among HIV-infected pregnant women. The SMS-based CD4 result distribution platform led to considerable improvement in time to clinic receipt of results and was associated with reduced operational costs. The intervention was well-received by clinic staff who perceived that it assisted in their care of HIV-infected pregnant women. However, the overall intervention was not associated with clinical or performance improvement as measured by time to CD4 phlebotomy or ART initiation.

The intervention was designed around the premise that early CD4 testing, prior to 26 weeks gestation, was critical to timely ART initiation in pregnancy. Findings from this trial support the notion that early CD4 testing is important (but not sufficient) for achieving this goal, with women having a CD4 draw prior to 26 weeks more than 7 times more likely to start ART prior to 30 weeks gestation. Unfortunately, only just over half of ART-naïve HIV-infected women underwent phlebotomy by 26 weeks, and there was no significant difference in time to CD4 draw between Intervention and Usual Care. It is unclear why the intervention would have contributed to the observed significant decrease in the odds of having a CD4 measured before delivery. While the SMS component of the intervention would not be expected to modify CD4 phlebotomy, the automated platform permitted real-time monitoring of testing volume and interventions to ensure stable access to testing supplies and re-education of clinic staff on the importance of CD4 testing at the first antenatal visit when testing volume dropped.

The second core premise of the intervention was that improving reliability and speed of CD4 result return to the antenatal clinic would accelerate the process of ART initiation in pregnancy. The SMS-based platform succeeded in reducing time to CD4 result receipt by the clinic and directing the result to the healthcare worker in the best position to act on the result. In addition, comments from clinic staff indicate that the intervention assisted with contacting and engaging ART-eligible pregnant women. However, with improving this aspect of the cascade, new obstacles to timely ART initiation were exposed. While we did not have access to utilization data at the ART clinics, interviews with antenatal staff and patients indicated that travel to the separate ART clinic and requirements for multiple visits for counseling and baseline laboratories were barriers, as has been observed elsewhere [32,33]. In the limited number of interviews of ART-eligible women who did not start ART, the need to complete counseling along with a friend or family member serving as an adherence partner was the most frequently cited reason for not starting ART in pregnancy. The intervention did not include activities facilitating care at the ART clinic or assisting with the disclosure of HIV status to a potential adherence partner.

Applications of mHealth technologies have greatly expanded and have been cited as an asset to public health programs in resource limited settings [34]. Like in this trial, many emerging applications of mHealth are employed to circumvent physical or operational barriers in strained healthcare systems. Prior successful uses of SMS for healthcare delivery have also focused on improving communication of clinical information across providers [24]. However, the overall evidence in support of mHealth applications to improve healthcare delivery in resource-constrained settings is limited with few interventions evaluated rigorously for effectiveness [35].

While direct-to-patient SMS programs has been shown in some settings to improve adherence to ART[23,25,36,37], to our knowledge, this is the first randomized trial of an mHealth-based intervention focusing on improving the cascade of activities necessary between HIV testing and ART initiation. Our negative findings, therefore are important and should provide valuable information to programs considering projects similar to those tested in this trial. It highlights both the importance of systematic evaluation of mHealth programs against endpoints that are clinically relevant, and the limited ability of these technologies to overcome persistent structural deficiencies in health systems. In our case, SMS messaging was an efficient and effective solution for reducing time between CD4 testing at a central laboratory and receipt of results at peripheral clinics. However, the intervention alone was not sufficient to address the many complex structural and behavioral barriers to ART initiation among pregnant women. Our study is an example of how mHealth interventions can play an important role as part of collaborative design frameworks, and how to evaluate interventions using discrete, programmatically relevant outcomes of interest [38,39]. Future interventions that include more robust programs to address remaining behavioral, educational, and clinic-based challenges to access may be more successful.

Promising alternatives to improving access to timely antenatal ART include strategies that reduce the number and simplify the steps of the prevention of MTCT cascade. These include point-of-care CD4 testing [40], decentralization of services to minimize transportation barriers [41,42], and minimizing or removing repeated adherence counseling visits [32]. Such programmatic changes have proven effective in scenarios like that recently established in Malawi that initiate ART without measuring CD4 count or other baseline laboratory tests [43]. Another promising strategy is to build longitudinal relationships with community-based lay health workers who help navigate the cascade [44].

The rapid turnaround of CD4 tests—as well as other critical laboratories—remains an important goal for ART programs throughout the developing world. In Botswana and other Option B programs, a CD4 result is still required prior to starting ART to inform whether to discontinue ART after delivery (or after breastfeeding) for women with CD4 greater than 350 cells/μL. CD4 enumeration remains important to clinical management in Option B+ and during routine ART treatment. Additionally, reliable receipt of early infant HIV PCR results, baseline or monitoring chemistries, cervical cancer screening results, and tuberculosis diagnostics are large challenges for many HIV treatment programs. The SMS-based platform demonstrated in this project successfully addressed laboratory turnaround and could have role to improving systems. However, the findings of this study suggest that this implementation needs to be tied to addressing remaining and emerging gaps in the health system.

The findings of this trial should be interpreted in the context of several important limitations. The maternity data extraction captured information from fewer women than anticipated and, following adjustment for clustering, the resulting confidence intervals for the effect estimates are wide. While the evidence from the primary and secondary analyses consistently argues against a beneficial effect, due to the wide confidence intervals we cannot exclude a clinically relevant difference. By chance, two large clinics serving a less educated population and offering early universal ART were randomized to be the first pair to receive the intervention. Consequently, the groups of women registering under Usual Care and under Intervention differed in some baseline characteristics indicating possible bias. Despite multivariate adjustment for these factors residual confounding is possible, although sensitivity analyses excluding these clinics did not modify findings. In addition, some contamination between study groups likely occurred with women receiving care from more than one ANC, or ANCs in transition from usual care to intervention, and clinicians rotating between different ANCs. However, different study group definitions did not modify the findings and the most successful aspect of the intervention—the automated SMS-platform—could only be accessed at intervention antenatal clinics. Also, due to staged design only clinicians at intervention sites were aware that clinic performance was being studied, possibly leading to increased motivation to improve care and overestimating the positive effects of the intervention (i.e. Hawthorne effect). Although our results are most applicable to Botswana, the demonstration that rapid CD4 resulting to peripheral clinics is achievable through an SMS-based system is likely to be applicable to many low- and middle-income country settings.

In conclusion, we found that a low-cost intervention involving clinic staff education and a novel SMS-based CD4 result delivery and monitoring platform did not significantly improve the proportion of women with antenatal CD4 testing by 26 weeks gestation or ART initiation by 30 weeks gestation. However, this intervention did result in more rapid and less expensive CD4 resulting to peripheral antenatal clinics. This SMS-based platform may improve the speed and reduce the cost of laboratory reporting in other clinical contexts, particularly in resource limited settings.

## Supporting Information

S1 CONSORT Checklist. (PDF)

S1 Protocol Trial protocol. (PDF)
