# Supplementary material for: An Augmented SMS Intervention to Improve Access to Antenatal CD4 Testing and ART Initiation in HIV-Infected Pregnant Women: A Cluster Randomized Trial
Source: PLoS One. 2015 Feb 18;10(2):e0117181. doi: 10.1371/journal.pone.0117181 (PMC4334487; doi:10.1371/journal.pone.0117181)
Supplement: S1 Protocol — (PDF) [file pone.0117181.s002.pdf]

BOTSWANA
HARVARD AIDS INSTITUTE
PARTNERSHIP
FOR HIV RESEARCH AND EDUCATION

Clarification Memo #1

DATE: 13 March 2014

SUBJECT: Clarification Memo #1 to the Tokafatso Protocol, Version 2.0, 14 December 2011 entitled “Programmatic CD4 Testing and HAART initiation among HIV-infected pregnant women in Gaborone, Botswana: a randomized staged trial of an improvement intervention”

This clarification memo does not result in a change in the protocol informed consent nor affect the risks and benefits for study participation.

Clarification to Tokafatso (BHP044) Protocol, Version 2.0, 14 December 2011:

1. Section 6, under the heading, Methods, provides general instructions for the implementation of the intervention in the study clinics. Further detail is provided below:

   Following at least 6 months of baseline data collection (conducted by the Birth Outcomes Surveillance Project team), the study period begins and continues until all enrolled clinics have received the intervention (approximately 12 months). During this period, two clinics will receive the Tokafatso intervention every month as described and diagrammed in funded and IRB-reviewed grant proposal (page 3). At the end of the study period all clinics will be operating under the Tokafatso intervention. Order of implementation will be randomized to achieve a 1:1 ratio of Intervention and Usual Care clinic-months during the course of the 12-month study period. Date of actual implementation of Tokafatso intervention procedures will be directed by conditions in the clinic. However for purposes of analysis the date for each new block (set of 2 clinics receiving the intervention) will be scheduled date of implementation determined prior to randomization.

2. Section 6.2, under the heading, Randomization Procedure, describes framework for clinic randomization. Further detail of procedures are provided below:

   Goal of clinic randomization is to achieve a 1:1 ratio of clinic-months between Intervention and Usual Care. Prior to the initiation of the study period clinics will be randomly assigned (using computerized random number generator) to ordered implementation pairs. This allocation will be concealed from implementation team and from clinic staffs. Clinic names assigned to each ordered implementation pair (e.g. 1st pair, 2nd pair, .. n^{th} pair) will be placed in sealed envelopes. One week prior to the scheduled pair implementation date, the labeled envelope will be opened revealing the names of the next implementation pair.

The Tokafatso project was implemented as described above, and as reflected elsewhere in the protocol, clinicaltrials.gov, and the IRB-approved protocol summary. This memo is a clarification of study procedures for the record.

Private Bag BO 320
Gaborone, Botswana
Phone: +267 -3902671
Fax: +267 -3901284
Website: www.bhp.org.bw

Programmatic CD4 Testing and HAART initiation among HIV-infected pregnant women in Gaborone, Botswana: a randomized staged trial of an improvement intervention

Botswana-Harvard AIDS Institute (BHP)
Private Bag BO 320, Bontleng
Gaborone, Botswana
Tel: 3902671, Fax: 3901284

1 Study sites and management

1.1 Study sites (BHP)

Gaborone

1.2 Management

All questions concerning this protocol should be sent via e-mail to scott_peterson@post.harvard.edu.

2 Abstract/Schema

TITLE	Programmatic CD4 Testing and HAART initiation among HIV-infected pregnant women in greater Gaborone, Botswana: a randomized trial of an improvement intervention
OBJECTIVE	The primary aim is to evaluate the efficacy of a clinic improvement intervention (daily CD4 specimen draws, facilitated transport, and active follow-up) towards improving rates of CD4 testing and HAART initiation among eligible HIV-infected pregnant mothers.
DESIGN	Cluster randomized study of clinics
METHODS	Clinics providing antenatal care in greater Gaborone will be randomized to either receive an improvement intervention immediately or receive improvement package after 4 months. The improvement intervention will include the capacity for daily CD4 specimen draws, facilitated transport, and active follow-up. Timing of maternal CD4 results and HAART initiation will be abstracted from obstetric records of women delivering at and Princess Marina Hospital (Gaborone). Proportion of women with CD4 testing prior to 26 weeks gestation and proportion of women with CD4+ cell counts less than 250 cells/µL who initiate HAART prior to 30 weeks gestation will be compared between the arms.
DURATION	Baseline data collection for 6 months, followed by randomized intervention for 6 months, followed by intervention in all clinics for 6 months. Total of 18 months.
SAMPLE SIZE	All busy antenatal clinics in greater Gaborone (20clinics) will be included. Approximately 1400 HIV-infected pregnant women not yet on HAART will be cared for in these clinics during the study period.

POPULATION Clinics serving HIV-infected pregnant women in greater Gaborone.

3 Specific Aims and Hypotheses

3.1 Primary Aims

1) Compare the proportion of HAART-eligible (CD4 <250) pregnant women who are started on HAART prior to 30 weeks gestation between clinics following usual care and those randomized to receive the improvement intervention.
Hypothesis: Pregnant women registering at clinics receiving the implementation package will have a 2-fold greater rate of HAART initiation before 30 weeks gestation (0.3 to 0.6).

2) Compare the proportion of HIV infected pregnant women who have a CD4 test result prior to 26 weeks gestation between clinics following usual care and those randomized to receive the improvement intervention.
Hypothesis: Clinics receiving the implementation package will have a 1.5-fold greater rate of CD4 testing prior to 26 weeks gestation (0.3 to 0.6).

3) Compare gestational age at CD4 testing and HAART initiation between patients cared for in clinics following usual care and those randomized to receive the improvement intervention.
Hypothesis: Patients seen at intervention clinics will have CD4 testing and HAART initiation earlier in pregnancy.

3.2 Secondary Aims

1) Compare the proportion of HAART-eligible (CD4 <250) pregnant women who are started on HAART prior to 30 weeks gestation in randomly selected clinics in the six months prior to the implementation of an improvement intervention and the six months following the intervention.
Hypothesis: Mothers registering at an intervention clinic after the implementation of an improvement package will have a 2-fold greater rate of HAART initiation before 30 weeks gestation than those registering during 6 months prior to implementation (0.3 to 0.6).

2) In an exploratory analysis, compare proportion of infant HIV-infections (at 6 weeks DNA PCR) born to HAART-eligible mothers registering at intervention clinics with those registering usual care clinics.
Hypothesis: Infants born to HAART-eligible mothers registering at intervention clinics will have an approximate 3-fold greater risk of HIV infection (2 versus 6 percent).

3) Explore relationship of possible predictors (education level, primagravida vs. other, age, occupation, HIV diagnosis during pregnancy vs. prior to pregnancy, gestational age at booking) of CD4 testing prior to 26 weeks or start of HAART prior to 30 weeks gestation.
Hypotheses: Women with prior pregnancies, HIV diagnosis prior to current pregnancy, and greater education will undergo CD4 testing and HAART more quickly.

4 Background and Rationale

Each year, more than 2 million children are born to HIV-infected women[1]. Despite

knowledge of effective approaches to nearly eliminate mother-to-child transmission (MTCT) [2, 3], worldwide infant HIV infection remains common. In 2008, is it estimated that 480,000 infants acquired HIV perinatally with over 90 percent of these infections occurring in sub-Saharan Africa[4]. Women with more advanced disease, with lower CD4 cell counts and peripheral HIV virus loads, are most likely to transmit HIV to their babies. In addition, these women with low CD4+ cell counts are at increased risk of HIV-related morbidity and mortality. Provision of highly-active antiretroviral therapy (HAART) to pregnant women, particularly those with low CD+ cell counts can ameliorate both risks[2, 5-9].

Motivated to improve maternal and infant health, and reduce the number of infant HIV infections, the Botswana National HIV/AIDS Treatment Guidelines prioritizes HAART-initiation for eligible pregnant women (CD4+ cell counts of less than 250 cells/μL or an AIDS event)[10]. Using a rapid HIV tests and lay counselors in antenatal clinics (ANC), over 95 percent of women Botswana presenting to ANCs undergo HIV testing and over 90 percent of HIV-infected women who are citizens of Botswana (with proof of citizenship) access medications for the prevention of MTCT[11]. For women eligible and participating in the national program for prevention of MTCT, the rate of HIV infection of their infants is low, estimated in 2008 at 3.6 percent[11].

Despite good access to some MTCT interventions, evidence suggests that access and uptake of HAART to eligible pregnant women is more limited. In a systematic review of records for women delivering at Princess Marina Hospital, the nation's busiest obstetric hospital, only half of HIV-infected women had a CD4+ cell count during pregnancy. Of women who had a CD4+ cell count and were eligible for HAART, only 37 percent were initiated on HAART prior to delivery[12]. Failure to initiate HAART in these eligible women is estimated to account for 20-30 percent of all infant infections in Botswana. It is also likely to contribute to excess maternal morbidity and mortality.

Barriers to CD4 testing and HAART initiation in Botswana have not been previously systematically evaluated. Preliminary data, drawn from recruitment statistics for clinical trials, suggest that newly diagnosed HIV-infected women presenting for antenatal care face considerable delays in CD4 testing and receipt of results (see preliminary data below). Additionally, many pregnant women change antenatal clinics or are otherwise lost-to-follow-up and CD4 results cannot be used in their clinical care. Barriers in Botswana seem to be similar to those experienced elsewhere. In Zambia, inability to draw specimen for CD4 on day of registration, insufficient transportation resources, and rural location were all associated with decreased CD4 testing[13].

To meet Botswana's stated goal of no new infections by 2016[14], further reductions in MTCT are necessary. Improving the initiation of HAART among qualifying mothers can lead to significant decreases in MTCT. However, knowledge of effective practices to increase testing and HAART initiation are limited.

5 Preliminary Studies

During the recruitment process for the CTX Safety Study, the proposing investigators visited each of the public clinics offering antenatal services in Gaborone and Molepolole. Lay counselors, midwives, and other clinic personnel were questioned about processes and procedures. In addition, performance issues noted and those raised by clinic staff were

discussed with potential study participants on the maternity wards of SLH and PMH. While not a systematic study, these key informant interviews provide valuable perspective on the obstacles in these communities for CD4 testing and HAART initiation in pregnancy.

Significant barriers exist preventing timely CD4 testing and HAART initiation in HIV-infected pregnant women in Gaborone and Molepolole. Problems are varied between clinics, clinic-types, and communities however principal obstacles appear to be similar:

1. Poor communication between PMTCT, ARV, and ANC providers
   Contributing factors: Heavy clinic volume, rigid job roles and limited cooperation, limited oversight of documentation
2. Infrequent draw of CD4 specimen on day of HIV diagnosis
   Contributing factors: Limited phlebotomy hours, CD4 draw only on certain days due to transport limitations, inadequate staff for phlebotomy
3. Unpredictable return of CD4 results
   Contributing factors: Cumbersome transportation routes for samples/results, no "chain of custody for results", frequent lost or inadequate specimens, limited clinic follow-up for missing results
4. Frequent patients lost to follow-up
   Contributing factors: Mobile population, HIV stigma, limited tracing capacity
5. Frequent outages of HIV test kits or CD4 requisition forms
   Contributing factors: No clinic staff could describe a back-up system for testing, neighboring clinics also out of stock at same time

The improvement package provided in this study aims to work to overcome these obstacles by facilitating improved clinic-laboratory integration allowing draw of CD4 specimen on day of HIV diagnosis and return of CD4 results within 1 week. In addition, the tracing of HAART-eligible patients will be bolstered. Finally, an ombudsperson will be available to assist with overcoming unforeseen obstacles (expired phlebotomy tubes, HIV test kit outages, etc).

6 Methods

The study will follow a "staged roll-out" or "stepped-wedge" design[15] to evaluate the efficacy of the intervention. After a 6-month period of baseline data collection, clinics will be randomized (1:1) to early or deferred receipt of the improvement package. Clinics randomized to early intervention will begin implementing the improvement package immediately. Clinics randomized to delayed implementation will begin the improvement package 6 months after the early intervention clinics.

All interventions will be at the clinic level with no direct patient contact, other than facilitation of care, by the study staff or investigators.

Measurement of the efficacy endpoints will occur through the anonymous review of maternity records at SLH and PMH already ongoing in the Birth Outcomes Study (has received ethical approval from Health Research and Development Committee, Botswana Ministry of Health and the IRB of the Office for Health Research Administration, Harvard School of Public Health). The Birth Outcomes dataset will contain the needed information to evaluate efficacy of the improvement package. No new data collection will be performed for the purposes of this study.

6.1 Determine Clinic Characteristics and Participation

All clinics providing antenatal services in Gaborone (including adjacent communities) will be visited. Clinic characteristics will be noted (including number of new ANC visits monthly, staff numbers, transportation availability, phlebotomy hours, laboratory facilities, presence of ARV clinic/pharmacy, adherence to registers, perceived and measured CD4 turn-around time, and phone access). Approval for participation in the study will be sought from the clinic leadership. Non-willing clinics will be excluded from the study.

6.2 Randomization Procedure

Participating clinics will be selected at random (1:1) to receive either immediate or delayed receipt of the improvement package.

6.3 Improvement Package

The improvement package will consist of 3 principal elements— expand access to CD4 testing, expedited return of CD4 results, and active follow-up of HAART-eligible women. Modification and additions may be necessary during the course of the study to meet changing challenges.

6.3.1 Expand access to CD4 specimen draw at first ANC visit

In our pilot data, women who are able to access CD4 testing on their first ANC visit receive their results a median of 1 month prior to those that do not. These women also have a greater likelihood of starting on HAART prior to delivery. Restrictive phlebotomy hours (due largely to constraints regarding transportation of specimens) prevent many women from having CD4 specimen drawn on the day of their first ANC visit.

The current standard of care is to only draw CD4 specimens in the morning of days when the clinic can transport specimens "same-day" to the referral laboratory. Depending on availability of transportation, some clinics offer CD4 testing 1-4 days per week. In addition all clinics are currently only allowing CD4 specimens to be drawn in the morning, so women who present or who are attended to after 10 or 11am cannot have a CD4 specimen drawn.

As part of the improvement package, each clinic will expand phlebotomy times to at least 4 days per week and during all clinic hours. This will be enabled through improved integration of clinic and laboratory services. Valid CD4 measurements can be achieved from specimens processed up to 72 hours after collection. Through use of priority labeling and communication with the laboratory, specimens can be drawn and kept (at room temperature) for 1 to 2 days (maximum duration to depend on requirements of the laboratory) in the clinic awaiting available transportation to the laboratory. These specimens would be processed in an expedited manner to allow CD4 measurement within the 72-hour window of validity.

6.3.2 Expedited return of results, chain-of-custody

Under current conditions, CD4 test results return to clinic sites via a variety of means and a variety of schedules. In some sites (Gaborone ARV clinics) results return electronically within days, however performance is limited by frequent system downtimes, computer malfunctions, and outages of consumables (printer toner and paper). In other sites

(remaining Gaborone clinics) clinic staff travel once every 2-4 weeks to receive CD4 test results from ARV clinic and transcribe them into patient registers. In other sites (all Molepolole, Tlokweng, and Mogoditshane sites), test results return in printed form via stretched transportation networks. In all settings, return of results is unpredictable so patients are often told to return weeks later to ensure results have returned. And if results do not return, it is not possible to audit where in the system the specimen was lost.

Under the improvement package, a plan will be developed for each clinic site so that results can be retrieved from the laboratory in less than 1 week. These plans will necessarily differ from clinic to clinic but will share a core set of objectives: 1) an audit chain of custody of specimen and results, 2) a rigorous clinic-based register to record PMTCT CD4 specimens and their results in and out of the clinic, 3) result return at least twice per week, and 4) deployable back-up system to that will continue to ensure return of results in less than one week in settings of computer/system outages, loss of transportation (i.e. vehicle receiving service), or other obstacles to performance.

In most (or all) sites an automated, electronic system for returning results to the antenatal clinics will be implemented. Using existing BHP-authored scripts to direct validated CD4 test results from the FACSCalibur (and FACSCount) to national clinical databases (Meditech), results will be directed to a validated SMS distribution list. Each clinic will have a unique SIM and this number will be linked to all specimens drawn at that facility. Confirmed results will be sent to the paired SIM in an SMS printer onsite in the antenatal clinic. Clinic staff will confirm receipt (confirming that patient receives care in that clinic) by entering clinician code (permitting maintenance of "chain-of-custody"). SMS system will be used in analogous fashion to send reports of specimen receipt and rejection.

6.3.3 Active follow-up of HAART-eligible women

Under the current care standard, clinics and their staff have limited capacity to trace patients who do not return for test results. Nearly all clinics lack capacity to call mobile phones and few patients have landlines. Most clinics have the resources to trace on foot patients living near to clinics, but often patients do not attend the closest clinics to their residence out of stigma. And given the investment of time and energy, staff do not trace patients on a routine basis.

With the improvement package, contact details (physical address, two mobile numbers and names of trusted contacts) will be obtained from all HIV-infected women registering at ANC. Women will be counseled regarding importance of obtaining CD4 results in a timely fashion for their and their infant's health. They will be informed that they will be traced if they do not return for results.

Women with CD4<250 not returning for results within 1 week will be contacted (via mobile phone or home visit) and asked to return. Women will be re-contacted if they do not return to care after initial contact. A mobile phone, held by the study staff, can be used for contacting patients if the clinic lacks this facility.

Procedures for tracing will not differ for sites participating in the universal HAART pilot where all pregnant women, regardless of CD4 cell count, will be started on HAART. Only women with CD4<250 will have enhanced active follow-up.

6.3.4 Infant HIV PCR results

Under current care all infants born to mothers with HIV are routinely scheduled on the maternity wards for a PCR testing at 4-6 weeks of age. However, many women do not bring their infants for testing or do not receive test results.

As part of the study improvement package, infants born to mothers with CD4<250 will be traced using PMTCT registers and community tracing by the local clinics where needed to facilitate testing and return of test results.

These activities will depend on available resources.

6.4 Outcome measures and predictors

The endpoints of proportion of women CD4 tested and started on HAART by target gestational ages will be measured at the maternity wards of SLH and PMH. Data currently being collected for the Birth Outcomes project will be used to assess the efficacy of the improvement package. In addition, data on potential predictors of timely CD4 testing and HAART initiation will taken from this dataset.

Specific data to be obtained from this surveillance dataset includes: demographics, clinic of ANC registration, date of ANC registration, last menstrual period, estimated date of delivery, date of delivery, parity, date of HIV test, date of CD4 test, and date of AZT or HAART initiation.

Brief, structured interviews will be performed on the maternity wards for HAART-eligible women and HIV-infected women without a recorded CD4 cell count, with their informed consent. Interviews will be used verify details (CD4 testing and HAART initiation dates) recorded in antenatal and maternity record. A brief descriptive narrative will be collected from these women. Women without a CD4+ cell count during the index pregnancy will be offered CD4+ testing, as per Botswana guidelines. Permission will be sought from HAART-eligible women to help facilitate (telephone or home visit) recommended and standard-of-care HIV testing for their infant(s), and abstraction of results from clinical records.

7 Study Management

7.1 Data Management

All data management for the core outcome and predictor variables will be performed by the Birth Outcomes Study. These procedures are described in detail below (taken from Birth Outcomes protocol).

7.1.1 Surveillance system

Surveillance data from obstetrical and medical records extracted at maternities will use either the maternal PM number or a unique study identification number created at the time of extraction, and this will be based upon the infant date of birth, maternal age, and infant birth weight. Mother and infant data will be collected on the same form. The PM number is a one-time assigned number and isn't available when the computers go down. These numbers cannot be used to link to maternal names or identifiers.

Extraction forms will be copied at each hospital location and the copies will be stored in a locked file cabinet (and destroyed at the end of the study). Originals will be transferred by driver or by FedEx to the BHP office in Gaborone, and received by a dedicated study data manager. Data will initially be entered into an Excel spreadsheet. On a weekly basis, the spreadsheet will be updated and maintained in one master database. All spreadsheet entries will be backed up and the master spreadsheet will be backed up upon creation each week. The spreadsheets will entered and maintained on the BHP computer server which has a high level of security and automated daily backup procedures in place. Approximately once every 2 months, the master spreadsheet will be electronically transmitted to the PI or statistician for import into a SAS analysis database.

7.1.2 Data storage

Paper forms will be maintained by the study data manager in locked cabinets in the locked room at BHP, and destroyed at the end of the study period after the final database is created. Copies will be kept in locked cabinets filed by study identification number at surveillance sites and destroyed at or before the end of the study.

Electronic data will be maintained by BHP researchers for up to 10 years. This database will be made available to the Botswana government and with other collaborating researchers performing IRB-approved projects, once the initial objectives of the study have been met. The database is used by permission of the Botswana government, which ultimately "owns" the data. The database may be used in the future by the Botswana government, and for other approved research as designated by the Botswana IRB and the Ministry of Health. No personal identifiers will be collected or maintained in this database.

7.1.3 QA/QC Procedures

QA/QC procedures for data collection and adherence to study protocol will be developed at the start of the study, and modeled on the extensive QA/QC procedures employed by previous BHP studies.

8 Human Subjects

8.1 Characteristics of study population

The study population will consist of clinics caring for HIV-infected pregnant women and their infants. As the study intervention takes place at the level of the clinic rather than at the individual, the most patients will not have contact with study investigators/staff. A subset of patients, mothers without recorded CD4 counts or who are HAART-eligible, will have contact with study staff.

8.2 Sources of research material

Research material will consist of data obtained by the Birth Outcomes study from obstetrical and neonatal records for all mother-infant pairs in surveillance. Validation interviews will be documented on case report forms.

8.3 Participant recruitment and study consent

Birth Outcomes study collects data by reviewing logbooks and delivery records. These data are collected without recording identifying information and without individual consent, as currently approved by Botswana and Harvard IRBs. Contact with individual patients within

the antenatal clinics will not be made (unless expressly requested by clinic staff) by study staff.

A sub-set of mothers, HAART-eligible or without a recorded CD4 count, will be approached on the maternity wards of the study hospitals. The purpose for the validation interviews will be described and patients will be asked to provide written informed consent. For ill or tired patients, interviews will be delayed until an appropriate time.

8.4 Risks and Benefits

The study serves to augment care currently being provided and does not pose a risk to individual patients cared for in participating clinics. As in any clinical care setting, there are risks associated with accessing health care (breach of confidentiality, complications of phlebotomy) however these risks are not expected to be modified by study activities. It is extremely unlikely that the improvement package will worsen the current standard of care.

It is possible that the employment status of staff working in clinics found to be poorly performing could be jeopardized. However this risk will be minimized by not identifying individuals or clinics in relation to performance. The identity of participating clinics will be kept confidential in all written reports and correspondence.

Both the clinics and the patients have the potential to benefit by study activities. Clinic staff will benefit from increased training and support. Patients, it is hoped, will be obtained improved care with more timely CD4 testing and HAART initiation.

8.5 IRB Review Plan

IRB approval, and annual review as required, will be obtained from Harvard School of Public Health, and the Botswana HRDC.

9 Analysis and Statistical Considerations

9.1 Endpoints

There are two primary endpoints: 1) CD4 testing prior to 26 weeks gestation (yes/no), and 2) HAART initiation prior to 30 weeks gestation (yes/no). For the purposes of these endpoints gestational age will be based on last menstrual period. In rare instance where the estimated date of delivery is modified by the midwife or doctor caring for the patient (based on ultrasound or other information) gestational age will be calculated from the estimated delivery date.

9.2 Analyses

Descriptive statistics (median and IQR and proportions) will be used to describe the clinics and the populations they serve. Descriptive statistics (proportions with 95% confidence intervals) will be used for patients having CD4 testing prior to 26 weeks and those starting HAART prior to 30 weeks gestation.

Statistical testing of proportions will done using Fisher's exact test (2-side with $\alpha = 0.05$), incorporating the clustered design. Testing of whether CD4 testing occurred earlier/later between the intervention arms will use the Wilcoxon Rank Sum non-parametric test.

For pre/post comparisons, paired procedures for of non-independent data will be used (McNemar's Test will be used for proportions and a Wilcoxon Signed Rank will be used for continuous data (gestational age at testing or initiation)

9.3 Power

Methods to estimate power for planned dichotomous comparisons for clustered data resulting from a "stepped wedge" study have been described in detail by Hussey and Hughes[16]. Utilizing baseline estimates of clinic-specific CD4 testing and HAART initiation performance (and measured coefficients of variation) drawn from the ongoing Birth Outcomes study (May 2009 to March 2010), we have used their methods to estimate the power of our planned comparisons for various effect sizes, Table 1. We have greater than 80% power for our primary comparisons given hypothesized intervention effect.

Table 1. Estimated power (α=0.05) for various effect sizes.

Endpoint	Est. Total Sample Size	Proportion Achieving Endpoint		Power
		Control	Intervention	
CD4 < 26 weeks GA	900	36%	46%	57%
CD4 < 26 weeks GA	900	36%	51%	90%
HAART<30 weeks GA	126	33%	58%	64%
HAART<30 weeks GA	126	33%	63%	79%
HAART<30 weeks GA	126	33%	68%	90%

10 Vertebrate Animals

Not applicable.

11 References

1 Towards universal access: scaling up priority HIV/AIDS interventions in the health sector: progress report, April 2007. World Health Organization, UNAIDS, UNICEF, Geneva 2007.

2 **Shapiro, R. L., Hughes, M., Ogwu, A., Kitch, D., Lockman, S., Moffat, C., Makhema, J., Moyo, S., Thior, I., McIntosh, K., van Widenfelt, E., Leidner, J., Powis, K., Ashmelash, A., Tumbare, E., Zwerski, S., Sharma, U., Handelsman, E., Mburu, K., Jayeoba, O., Moko, E., Souda, S., Lubega, E., Akhtar, M., Wester, C., Snowden, W., Martinez-Tristani, M., Mazhani, L. and Essex, M.,** A Randomized Trial Comparing Highly Active Antiretroviral Therapy Regimens for Virologic Efficacy and the Prevention of Mother-to-Child HIV Transmission among Breastfeeding Women in Botswana *5th IAS Conference on HIV Treatment, Pathogenesis and Prevention*, Cape Town, South Africa 2009.

3 **Dao, H., Mofenson, L. M., Ekpini, R., Gilks, C. F., Barnhart, M., Bolu, O. and Shaffer, N.,** International recommendations on antiretroviral drugs for treatment of HIV-infected women and prevention of mother-to-child HIV transmission in resource-limited settings: 2006 update. *Am J Obstet Gynecol* 2007. **197**: S42-55.

4 **UNAIDS,** AIDS epidemic update : November 2009. UNAIDS, Geneva 2009.

5 **Arendt, V., Ndimubanzi, P., Vyankandonera, J., Ndayisaba, G., Muganda, J., Courteille, O., Rutanga, C., Havuga, E., Dhont, N., Mujawamassiga, A., Omes, C. and Peltier, A.,** AMATA study: effectiveness of antiretroviral therapy in breastfeeding mothers to prevent post-natal vertical transmissionin Rwanda *IAS*, Sydney, Australia 2007.

6 **Kilewo, C., Karlsson, K., Ngarina, M., Massawe, A., Lyamuya, E., Lipyoga, R., Msemo, G., Swai, A., Mhalu, F. and Biberfeld, G.,** MITRA Plus: Prevention of mother-to-child transmission for HIV-1 through breastfeeding by treating mothers prophylactically with triple antiretroviral therapy in Dar es Salaam, Tanzania *4th IAS Conference on HIV Pathogenesis, Treatment, and Prevention*, Sydney, Australia 2007.

7 **Palella, F. J., Jr., Deloria-Knoll, M., Chmiel, J. S., Moorman, A. C., Wood, K. C., Greenberg, A. E. and Holmberg, S. D.,** Survival benefit of initiating antiretroviral therapy in HIV-infected persons in different CD4+ cell strata. *Ann Intern Med* 2003. **138**: 620-626.

8 **Sterling, T. R., Chaisson, R. E., Keruly, J. and Moore, R. D.,** Improved outcomes with earlier initiation of highly active antiretroviral therapy among human immunodeficiency virus-infected patients who achieve durable virologic suppression: longer follow-up of an observational cohort study. *J Infect Dis* 2003. **188**: 1659-1665.

9 **Emery, S., Neuhaus, J. A., Phillips, A. N., Babiker, A., Cohen, C. J., Gatell, J. M., Girard, P. M., Grund, B., Law, M., Losso, M. H., Palfreeman, A. and Wood, R.,** Major clinical outcomes in antiretroviral therapy (ART)-naive participants and in those not receiving ART at baseline in the SMART study. *J Infect Dis* 2008. **197**: 1133-1144.

10 **Botswana Ministry of Health,** Botswana 2008 National HIV/AIDS Guidelines. Ministry of Health, Botswana 2008.

11 **Gaolathe, T., Tlale, J., Keapoletswe, K., Anderson, M. G., de la Hoz Gomez, F., Mmelese, M. and Seipone, K.,** Mother-to-Child HIV Transmission Rate in Botswana - Analysis of Dried Blood Spot (DBS) Results from the National PMTCT Programme *4th IAS Conference on HIV Treatment, Pathogenesis and Prevention*, Mexico City 2008.

12 **Chen, J. Y., Ogwu, A. C., Svab, P., Lockman, S., Moffat, H. J., Gaolathe, T., Moilwa, S., Stordal, K., Dryden-Peterson, S., Moffat, C., Makhema, J., Essex, M. and Shapiro, R. L.,** Antiretroviral Treatment Initiation Among HIV-Infected Pregnant Women with Low CD4+ Cell Counts in Gaborone, Botswana. *J Acquir Immune Defic Syndr* 2009.

13 **Mandala, J., Torpey, K., Kasonde, P., Kabaso, M., Dirks, R., Suzuki, C., Thompson, C., Sangiwa, G. and Mukadi, Y. D.,** Prevention of mother-to-

child transmission of HIV in Zambia: implementing efficacious ARV regimens in primary health centers. *BMC Public Health* 2009. **9**: 314.

14 **Botswana, G. o.,** Vision 2016: Towards Prosperity for All 2001.

15 **Brown, C. A. and Lilford, R. J.,** The stepped wedge trial design: a systematic review. *BMC Med Res Methodol* 2006. **6**: 54.

16 **Hussey, M. A. and Hughes, J. P.,** Design and analysis of stepped wedge cluster randomized trials. *Contemp Clin Trials* 2007. **28**: 182-191.
